# Unlocking Salinity Stress Resilience in Turnip (*Brassica rapa* subsp. *rapa*) Plants Using *Bacillus subtilis* Z-12 and *Bacillus aryabhattai* Z-48

**DOI:** 10.3390/microorganisms13020359

**Published:** 2025-02-07

**Authors:** Imran Khan, Areeba Rehman, Waheed Akram, Tehmina Anjum, Nasim Ahmad Yasin, Zill-e-Huma Aftab, Bareera Munir, Waheed Ullah Khan, Guihua Li

**Affiliations:** 1 Guangdong Key Laboratory for New Technology Research of Vegetables/Vegetable Research Institute, Guangdong Academy of Agricultural Sciences, Guangzhou 510640, China; dh18006@yzu.edu.cn (I.K.); areebarehman453@gmail.com (A.R.); 2 College of Earth and Environmental Sciences, University of the Punjab, Lahore 54590, Pakistan; bareeramunir@yahoo.com (B.M.); waheed.cees@pu.edu.pk (W.U.K.); 3 Department of Plant Pathology, Faculty of Agricultural Sciences, University of the Punjab, Lahore 54590, Pakistan; waheedakram.fas@pu.edu.pk (W.A.); huma.dpp@pu.edu.pk (Z.-e.-H.A.); 4 Department of Horticulture, Faculty of Agricultural Sciences, University of the Punjab, Lahore 54590, Pakistan; nasimahmad.fas@pu.edu.pk

**Keywords:** PGPR, *Bacillus* spp., *Brassica rapa*, turnip, microbial inoculation, salt stress

## Abstract

Salinity stress poses a severe risk to food security and crop productivity. Stress reduction techniques are not necessarily sustainable or environmentally friendly. With the increasing adverse impact of salinity and area, it is necessary to restore and ameliorate salinity stress using environmentally friendly approaches. In this context, beneficial rhizospheric microbes may offer a sustainable approach to managing salinity stress. We used *Bacillus subtilis* strain Z-12 and *B. aryabhattai* strain Z-48 to improve the growth of turnip (*Brassica rapa* subsp. *rapa*) plants under salinity stress conditions and elucidated the beneficial impact of these bacterial strains on different physiological and biochemical aspects of plants. The application of both strains had a significant (*p* < 0.05) positive influence on analyzed parameters under salt stress. Here, *B. aryabhattai* strain Z-48 superiorly increased shoot length (33.2-, 25.8%), root length (38.6-, 31.5%), fresh biomass (23.9-, 17.8%), and dry biomass (38.60-, 48.6%) in normal and saline stress (200 mM NaCl) conditions, respectively. Physiological studies showed that antioxidant enzyme activities were significantly increased by *B. subtilis* Z-12 and *B. aryabhattai* Z-48 under salinity stress, with a few exceptions. Moreover, the inoculation of both strains effectively increased total chlorophyll, soluble sugar, phenolic, flavonoid, and glucosinolate contents under simulated salinity stress and normal conditions. Hence, these findings support the framework that inoculating turnip plants with these strains can enhance their tolerance against salinity stress.

## 1. Introduction

The increasing world population is accompanied by more food demand, and *Brassica* plants are essential to satisfy food needs. Turnip (*Brassica rapa* subsp. *rapa*) is an important vegetable crop cultivated in Asian countries due to its bulbous roots and fleshy leaves, consumed cooked or raw in different food recipes. Turnips are preferably cultivated due to their high biomass per hectare, starch content, and favoring long-term storage [1]. Among different glucosinolate compounds, gluconasturtiin, glucoraphanin, and glucobrassicin are abundantly found in turnips, famous for medicinal properties and chemopreventive activity [2].

Global crop production and agricultural yields are adversely affected by various environmental stresses that have emerged due to climate change [3]. Salinity is an essential environmental stress responsible for fertility degradation and poses a significant threat to global agricultural production. Approximately 12 million km^2^ of agrarian soil is affected by salinity stress worldwide [4], spanning 20% of agricultural lands worldwide [5]. Salinity damages plants by limiting water availability, causing high osmotic stress, and causing ionic toxicity [6]. The plant suffers a wide range of physiological and biochemical damages, such as reduced nutrient absorption, damaged photosynthetic machinery, ionic imbalance, and disruption in the biosynthesis of primary and secondary metabolites [6,7,8]. All of these factors severely affect plant growth and can cause up to a 50% reduction in agricultural yield [9]. Immediate, affordable, and sustainable solutions are sorely needed for salinity mitigation.

New effective techniques are vital in fostering agricultural production and food security under saline stress. Plant breeders are currently attempting to generate salt-tolerant varieties, but their accessibility is minimal. One approach to promote salinity tolerance in plants is using beneficial microbial inoculants. Using beneficial rhizospheric microorganisms is a sustainable method to counter the impact of salinity on crop yield and plant development [10]. These are important in providing abiotic stress tolerance and sustaining plant productivity, as the root microbiome is vital to plant hosts. The demand for microbial inoculants is increasing at an annual rate of 12% because of the high cost of chemical fertilizers and the desire for eco-friendly solutions [11]. The rhizosphere contains plant growth-promoting rhizobacterial strains (PGPR) that stimulate plant growth via several mechanisms [12]. PGPR promote the intake of nutrients by nitrogen fixation and access to minerals, iron, and phosphorus [13]. Apart from this, they also secrete exogenous substances such as bacteriocins, lipopeptides, hydrolytic enzymes, hydrogen cyanide (HCN), indole acetic acid (IAA), gibberellins, cytokinins, and siderophores to modify the hormonal status of the plant, which thus influences various in-built processes involving pathogen control, nutrient assimilation, and nutrient mobilization [14,15]. PGPR control the redox balance of salinity-affected plants by affecting the synthesis of osmoprotectants and antioxidant metabolites and by promoting polyamines [16]. All this would maintain water balance and stomatal conductance, stabilize subcellular structures, and increase photosynthetic efficiency [17]. The production of cation-binding exopolysaccharides (EPS) through PGPR limits plant salt intake and maintains ion homeostasis. In addition, EPS-producing PGPR increase the soil’s capacity to retain water and nutrients, thereby enhancing the water and nutrients available to the inoculated plants [18]. These mitigating mechanisms significantly improve plants’ physiological, biochemical, and morphological processes, such as photosynthetic efficiency, oxidative stress tolerance, and growth parameters [19].

PGPR are found to enhance resistance against abiotic stresses, and, simultaneously, they increase the ability of plants to survive under biotic stresses [20]. Previous studies have substantiated the importance of beneficial bacterial strains in reducing salinity stress in different crop plants. For instance, two bacterial strains, e.g., *Kocuria erythromyxa* and *Staphylococcu skloosii*, improved radish plant growth under saline stress by reducing membrane damage [21]. Similarly, *Bacillus oryzicola* YC007 increased plant growth attributes, including shoot length, biomass accumulation, and chlorophyll contents in *Raphanus sativus* and *Brassica oleracea* by regulating salinity-stressed-related pathways [22]. Bacterial strains belonging to *Bacillus cereus*, *Exiguobacterium aestuarii*, and *Bacillus megaterium* increased the growth of *Brassica juncea* plants under salinity and cadmium stress through reduced Na^+^ and Cd^++^ uptake in the shoots [23]. Additionally, applying *Azotobacter chroococcum* increased the growth of *Lactuca sativa* and *Raphanus sativus* seedlings raised under salinity stress [24].

Under salinity stress conditions, applying plant growth to promote beneficial microbes is preferable for enhancing crop productivity. Beneficial bacteria belonging to the *Bacillus* genus are predominantly found in the crop rhizosphere. Members of the *Bacillus* genus are metabolically and genetically diverse and can thrive under stressful environmental conditions due to the formation of stress-tolerant spores [25]. The *Bacillus* genus is preferred when developing microbial bio-fertilizers owing to its diverse plant-beneficial traits. Diverse genetic features in *Bacillus* enable their survival under adverse conditions. The presence of genes responsible for osmotic regulation, signal transduction, and antioxidant enzyme machinery contributes to the survival of *Bacillus* strains under salt stress [26]. Additionally, these bacteria regulate the stress signaling molecules inside the plant body to alleviate stress conditions in plants [26]. *Bacillus* spp. secrete several metabolites trigger plant growth and enhance plant tolerance to biotic and abiotic stresses [27]. To ameliorate the impact of salinity stress, *Bacillus* spp. use different mechanisms, such as inhibiting the reactive oxygen species formation accumulation of phytohormones, osmolytes, and other biomolecules inside the plant body [27]. *Bacillus* curtails increased ethylene production in plants under salinity by ACC-deaminase-mediated hydrolyzation activity [28]. *B. subtilis* improved the growth of *Trigonella foenumgraecum* by decreasing ethylene-induced damages during drought stress [29]. *B. subtilis* isolated from salt-affected soil increased wheat growth by releasing phytohormones IAA and gibberellin [30]. Abscisic acid (ABA) promotes plant growth and regulates water balance in plants during salinity stress [31]. The application of *B. subtilis* strain IB22 up-regulated the ABA biosynthesis gene in barley under salinity stress [32]. Firstly, the plant-beneficial effect of *Bacillus aryabhattai* was studied on *Xanthium italicum* [33]. *B. aryabhattai* increased the growth of wheat and soybean plants with the enhanced mobilization of nutrient elements [34].

However, there is a need to find competent microbial strains with superior properties and mechanisms underlying induced stress tolerance in plant systems. Bearing in mind the deleterious impact of saline stress on turnip crops and considering the importance of beneficial microbes in stress alleviation, we analyzed two PGPR strains, *Bacillus subtilis* (Z-12) and *Bacillus aryabhattai* (Z-48), to ameliorate salinity stress. The strains were selected based on their plant-growth-promoting and induced stress tolerance ability in our recent research work on tomato plants [35]. The study further intended to examine the mechanisms behind induced stress tolerance in the different physiological attributes of turnip plants. We investigated the effect of these bacterial strains on photosynthetic pigments, total soluble sugar, phenolic, and medicinally important glucosinolate content under normal and saline stress conditions.

## 2. Materials and Methods

### 2.1. Experimental Design

The turnip (*Brassica rapa* subsp. *rapa*) variety Purple Top White Globe, sold by Green Gold Seeds, Faisalabad, Pakistan, was used in this study. For surface sterilization, seeds were immersed in 70% ethanol for 30 s and 1% NaOCl for an additional 60 s, following multiple washes with distilled water. The seeds were planted in 12-inch-diameter plastic pots filled with commercial peat moss potting substrate (Rekyva, Šiauliai, Lithuania). The potting substrate was autoclaved before filling to ensure sterilization. Six seeds were sown in each pot, and the pots were kept in a wire house under natural daylight and temperature conditions. Three uniform plants were left in each pot after 7 d of emergence. Aqueous suspensions of each bacterial strain, e.g., *B. subtilis* strain Z-12 and *B. aryabhattai* strain Z-48, were prepared by growing them in Luria-Bertani (LB) broth media for 24 h. The next day, bacterial cells were obtained through centrifugation and mixed in sterilized water. The inoculum density was adjusted to 10^7–8^ CFU/mL using a spectrophotometer by observing an OD of 0.1 at 600 nm. The designated plants were given bacterial inoculation at 100 mL of culture suspension per pot. After one week of bacterial application, salinity stress (SS) was provided by irrigating the pots using only a solution of NaCl (200 mM) according to the experimental design. At the same time, the rest of the plants were irrigated with distilled, sterilized water. There were five treatments in the experiment: control (untreated control); SS (200 mM NaCl); Z-12 (*B. subtilis* strain Z-12 alone); Z-48 (*B. aryabhattai* strain Z-48 alone); SS + Z-12 (200 mM NaCl + *B. subtilis* strain Z-12); and SS + Z-48 (200 mM NaCl + *B. aryabhattai* strain Z-48). Each treatment consisted of nine replicate plants, and the whole experiment was repeated twice. The plants were harvested 60 d after emergence. Upon harvest, plants were gently washed to remove soil particles, and growth parameters were observed, including plant height, root length, fresh biomass, and dry biomass.

### 2.2. Estimation of Chlorophyll, Carotenoid, and Total Soluble Sugar Contents

Leaf material was crushed in liquid nitrogen, extracted (1 g) in 10 mL of 80% acetone, and centrifuged at 8870× *g* for 10 min. The OD of the supernatant was observed at 663- and 645 nm using a spectrophotometer. The quantities of chlorophyll and carotenoid contents were obtained using the equations [36].

Chlorophyll ‘a’ = (12.21 × OD654 − 2.81 × OD636)

Chlorophyll ‘b’ = (20.13 × OD639 − 5.03 × OD653)

Total Chlorophyll = (7.18 × OD645 + 17.32 × OD656)

Carotenoid = [1000 × OD469 − 3.27 × {(chl a − 104) × chl. b}]/229

### 2.3. Estimation of Total Phenolic, Flavonoid, and Glucosinolate Contents

Total phenolic content was estimated using the Folin-Ciocalteu reagent method by Singleton and Rossi [37]. Briefly, leaf material (1 g) was extracted in methanol (80%), and the reaction contained extract (0.5 mL), Folin-Ciocalteu reagent (0.5 mL), distilled water (8 mL), and aqueous 1 M Na_2_CO_3_ (1 mL). After one hour of incubation, absorbance at 765 nm was represented as mg GAE g^−1^ FW. A standard curve was produced using gallic acid.

Previously extracted plant material was used to quantify the total flavonoid content using the aluminum trichloride colorimetric method described by Chang et al. [38]. Rutin (10 to 100 µg mL^−1^) was used to create a standard curve, and absorbance was measured at 510 nm. The total flavonoid content was reported as mg Rutin g^−1^ FW compared with the standard curve.

The method of Kiddle et al. [39] was used to assess the total glucosinolate content. Fresh leaf material (1 g) was crushed in 5 mL of extraction solvent (70:30 methanol: water). The extract was then heated to 70 °C for 25 min to inhibit myrosinase activity. The extract was centrifuged for 15 min at 8000 rpm. Then, 3 mL of sodium tetrachloropalladate (2 mM) and 0.3 mL of double-distilled water were added to 100 µL of the extract. The mixture was incubated for 60 min, and OD was observed at 425 nm. The glucosinolate content was expressed as µmole g^−1^ FW.

### 2.4. Estimation of Antioxidant Enzymes

A total of 1 g of fresh leaf material was crushed in liquid nitrogen and extracted with 10 mL of 0.1 M phosphate buffer (pH 7.2). The mixture was centrifuged, and clear supernatant was used as an enzyme source. Briefly, Superoxide Dismutase (SOD) activity was analyzed using the reduction of Nitroblue Tetrazolium (NBT) using the method proposed by Dhindsa et al. [40]. The reaction mixture contained NBT (25 µM), sodium carbonate (50 mM), freshly made hydroxylamine hydrochloride (0.1 mM), and enzyme extract (100 µL). Following shaking, tubes were exposed to intense light for 15 min. The absorbance variations were recorded at 530 nm. The reaction mixture for the quantification of catalase (CAT) enzyme included 1 mL of 75 mM H_2_O_2_, 3 mL of phosphate buffer (0.1 M), and 0.5 mL of enzyme extract, according to Maehly [41]. The absorbance of the reaction was observed at 260 nm. To quantify the peroxidase (POX) enzyme, the reaction mixture consisted of 0.1 M H_2_O_2_, 0.25% guaiacol, 2.1 mL of 10 mM phosphate buffer, and 200 µL of enzyme extract. After 5 min of incubation, the OD at 510 nm was measured. The ascorbate peroxidase (APX) enzyme mixture consisted of 0.1 mM H_2_O_2_, 0.5 mM ascorbic acid, 100 µL enzyme extract, and 50 mM potassium phosphate buffer following the method by Nakano and Asada [42]. Absorbance was observed at 290 nm.

### 2.5. Statistical Analysis

The completely randomized design was followed during the experiments. Five biological replicates were included in each treatment, and experiments were repeated twice. The significant differences among treatments were calculated by performing ANOVA and Tukey’s HSD test at *p* < 0.05 using DSAASTAT software (Version 1.0192) developed by Onofri (Perugia, Italy). Origin 2018 software (Northampton, MA, USA) was used for correlation analysis.

## 3. Results

### 3.1. Effect of Bacterial Strains on Growth Attributes of Turnip Plants

**Figure 1:**
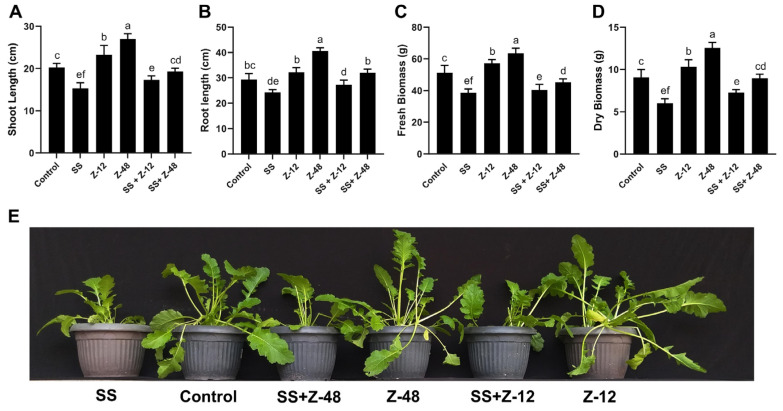
Effect of PGPR and salinity stress on different growth parameters of turnip plants (**A**–**D**). The treatments followed by the same letter are not significantly different at *p* < 0.05. Bars indicate the standard error of the means. Plant phenotype displaying the effect of salinity and PGPR treatments in various combinations (**E**). SS = salinity stress, Z-12 = *B. subtilis* strain Z-12, and Z-48 = *B. aryabhattai* strain Z-48.

Salinity substantially reduced growth traits such as plant height, root length, and biomass accumulation in *Brassica* plants (Figure 1). The application of bacterial strains significantly improved growth characteristics and ameliorated the adverse effects of salinity (Figure 1). Both the strains improved plant growth under saline and normal conditions, but *B. aryabhattai* Z-48 performed relatively better than *B. subtilis* Z-12 (Figure 1).

Under salinity stress, *B. aryabhattai* Z-48 increased shoot length (25.8%), root length (31.5%), fresh biomass (17.3), and dry biomass (48.6) compared to salinity stress control (SS) plants. Whereas, under normal conditions, shoot and root length, fresh biomass, and dry biomass were increased up to 33.2-, 38.6-, 23.9-, and 38.60% compared to the non-treated control (Figure 1). Similarly, under saline stress, *B. subtilis* Z-12 increased shoot length (13.5%), root length (12.2%), and dry biomass (20.6%) compared to the respective salinity stress control (Figure 1).

### 3.2. Effect of Bacterial Strains on Chlorophyll, Carotenoid, and Total Soluble Sugar Contents

Photosynthetic pigments, including chlorophyll a, b, total chlorophyll, and carotenoid contents, were significantly reduced in turnip plants raised under salt stress (SS) compared to non-treated control plants, as shown in Table 1. However, inoculation with bacterial strains *B. aryabhattai* Z-48 and *B. subtilis* Z-12 countered NaCl’s detrimental effects and enhanced chlorophyll content (Table 1). Under salinity stress, *B. aryabhattai* Z-48 and *B. subtilis* Z-12 resulted in an increase of 3.91- and 1.75-fold in Chl a and 2.48- and 1.73-fold in Chl b contents compared to the respective salinity stress control (SS) plants (Table 1). *B. aryabhattai* Z-48 and *B. subtilis* Z-12 increased total chlorophyll content by up to 3.24- and 1.61-fold in the same scenario (Table 1). *B. aryabhattai* Z-48 also significantly increased Chl a (2.54-fold), Chl b (2.06-fold), and total chlorophyll contents (2.35-fold) compared to the non-treated control in normal conditions (Table 1).

Under salt stress, plants showed a more considerable decrease in carotenoid content (2.54 fold) than non-treated control plants (Table 1). Under salinity stress, the application of *B. aryabhattai* Z-48 and *B. subtilis* Z-12 increased carotenoid content by up to 1.9- and 1.46-fold than the salinity control (Table 1).

The same was observed for soluble sugar content. Under salinity, there was a noticeable decrease (1.41-fold) in total soluble sugar than in the control plants (Table 1). The application of *B. aryabhattai* Z-48 increased soluble sugar content up to 1.82- and 1.39-fold under salinity stress and normal conditions compared with the respective controls (Table 1).

### 3.3. Effect of Bacterial Strains on Glucosinolate, Phenolic, and Flavonoid Contents

**Figure 2:**
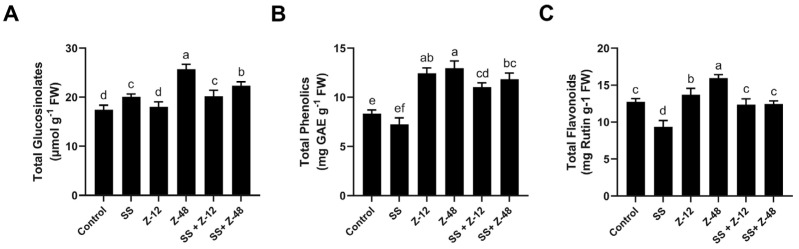
Effect of PGPR and salinity stress on total glucosinolates (**A**), total phenolic (**B**), and flavonoid content (**C**). The treatments followed by the same letter are not significantly different at *p* < 0.05. Bars indicate the standard error of the means. SS = salinity stress, Z-12 = *B. subtilis* strain Z-12, and Z-48 = *B. aryabhattai* strain Z-48.

From the data presented in Figure 2, it is evident that glucosinolate production in both normal and salinity stress was significantly affected by bacterial treatments (Figure 2). Interestingly, salinity stress increased glucosinolate content by up to 17.6% more than the non-treated control (Figure 2). *B. aryabhattai* Z-48 and *B. subtilis* Z-12 strains increased the yield of glucosinolate content by 33.1- and 12.7% in turnip plants under normal conditions (Figure 2). Similarly, in salinity stress (SS) conditions, *B. aryabhattai* Z-48 and *B. subtilis* Z-12 increased glucosinolate content by 11.3- and 5.9% compared to the respective salinity control (SS) treatment (Figure 2).

The application of *B. aryabhattai* Z-48 and *B. subtilis* Z-12 increased the total phenolics in turnip plants from 8.34 mg GAE/g FW (control) to 12.96 mg GAE/g FW (*B. aryabhattai* Z-48) and 12.45 mg GAE/g FW (*B. subtilis* Z-12), respectively, under normal conditions (Figure 2). Under salinity stress, total phenolic content increased up to 53.7- and 42.9% for *B. aryabhattai* Z-48 and *B. subtilis* Z-12, respectively, compared to the respective salinity stress control (Figure 2). Similarly, *B. aryabhattai* Z-48 significantly increased flavonoid content up to 32.9- and 24.5% under normal and salinity conditions, respectively, compared with the respective control treatments (Figure 2).

### 3.4. Effect of Bacterial Strains on Antioxidative Enzymes

The results of antioxidant enzyme activity analysis revealed that salinity stress considerably increased the activities of antioxidant enzymes compared to non-treated control treatment (Table 2). Here, the symbiosis of *B. aryabhattai* Z-48 and *B. subtilis* Z-12 further boosted enzyme activities and showed significant differences compared to salinity control (SS) treatment (Table 2). *B. aryabhattai* Z-48 provided significantly higher antioxidant activities, i.e., SOD (42.7%), CAT (7.9%), POX (153%), and APX (65.3%), than the salinity control (SS) treatment (Table 2). Similarly, *B. subtilis* Z-12 increased antioxidant activities, i.e., SOD (17.7%), CAT (0.6%), POX (63.9%), and APX (52.6%), compared to the salinity control (SS) treatment (Table 2).

### 3.5. Pearson Correlation and Principal Components Analysis

**Figure 3:**
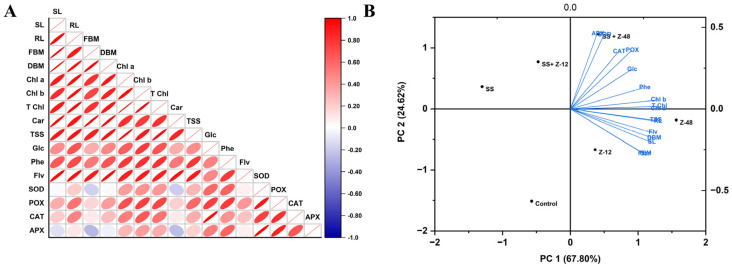
(**A**) Pearson’s correlation matrix based on morpho-physiological and biochemical parameters under the control, PGPR, and salinity stress conditions. Correlations are displayed in red (positive) and blue (negative); color intensity and eclipse size are proportional to the correlation coefficient. (**B**) The principal component biplot of a turnip plant is based on the variance of morpho-physiological and biochemical parameters under control, PGPR, and salinity stress conditions. The length of the arrows indicates the attributes’ contribution to the PCA components. SS = salinity stress, Z-12 = *B. subtilis* strain Z-12, and Z-48 = *B. aryabhattai* strain Z-48. SL = shoot length; RL = root length, FBM = fresh biomass; DBM = dry biomass; Chl a = chlorophyll a; Chl b = chlorophyll b; T Chl = total chlorophyll; car = carotenoid contents; TSS = total soluble sugar contents; Glc = glucosinolate contents; Phe = total phenolic contents; Flv = total flavonoid contents; SOD = superoxide dismutase; POX = peroxidase; CAT = catalase; APX = ascorbate peroxidase.

A Pearson correlation heatmap was created to examine the relationship between the factors under research (Figure 3). A noteworthy positive association existed between the observed growth characteristics, photosynthesis, and antioxidant machinery. Plant growth and photosynthetic characteristics were negatively correlated with the onset of salinity stress. The symbiosis of *B. subtilis* strain Z-12 and *B. aryabhattai* strain Z-48 mostly presented a positive correlation with plant growth attributes and antioxidant enzymes (SOD, CAT, POD, and APX). Additionally, total phenolic, flavonoid, and carotenoid contents showed positive correlations with the presence of *B. subtilis* strain Z-12 and *B. aryabhattai* strain Z-48, in either combination, indicating that these microbes play a beneficial function in enhancing growth and photosynthesis under salt stress (Figure 3).

The PCA scores used to assess how *B. subtilis* strain Z-12 and *B. aryabhattai* strain Z-48 affect turnip plants under salt stress are shown in Figure 3. PC1 and PC2 determined 92% of the variance in the dataset. PC1 supplied 67.8% of the overall variation, whereas PC2 contributed 24.6%. These main components successfully distributed the treatments. It is evident from the biplot that the antioxidants and photosynthetic machinery were situated between the plant growth-related traits, indicating their potential function in preventing salt stress. As a result, the correlation biplot analysis showed a strong correlation between bacterial strains and turnip plant salt acclimation (Figure 3).

## 4. Discussion

Salinity is a widespread environmental restraint adversely affecting agricultural productivity and food safety. According to an estimate, salinity causes 20–50% yield losses in agricultural produce, including grain and vegetable crops [43]. Therefore, there is a need for time to explore and adopt sustainable strategies, including the use of soil amendments, along with the cultivation of resistant crop varieties, to improve the yield and adaptation of plants in saline soils. Using beneficial microbes is an effective remedy to the current dilemma mentioned above. A range of beneficial microbes can rescue plant growth in saline soils [44]. Inoculated microorganisms have to compete with autochthonous microbial communities to persist in soils [45], which can affect their colonization and performance in field conditions. The de novo synthesis of osmoprotectants and ion transport systems enables them to survive in these extreme environmental conditions [46]. Despite *Bacillus* being the most studied plant growth-promoting genera under saline conditions [47], very few studies have been published on *B. aryabhattai* that deal with field crops [34]. To the best of our knowledge, this study is the first to report the beneficial effect of *B. aryabhattai* on *Brassica* vegetable crops and its underlying mechanisms.

Our study focused on exploring the beneficial impact of two bacterial strains belonging to the *Bacillus* genera on the growth enhancement of turnip plants under salinity stress through the modulation of biochemical and physiological mechanisms. These bacterial strains were previously isolated from the rhizosphere of plants cultivated in normal, non-saline fields. These strains proved their plant-beneficial properties by suppressing biotic and abiotic stresses in our previous studies using other crop plants [35].

Plants raised in saline soils exhibit different attributes in terms of growth and biochemical properties, resulting in reduced crop yield and quality. We observed a similar negative response on the growth attributes (shoot and root length, biomass accumulation, and photosynthetic and antioxidant machinery) of turnip plants raised under salinity stress. Physiological mechanisms, including decreased meristematic activity cell proliferation, can contribute to this adverse effect of salinity stress [48].

Previous studies have highlighted the importance of some bacterial strains belonging to the *Bacillus* genera in the amelioration of plant stress and growth promotion under adverse conditions. Oubaha et al. [49] reported a significant increase in rice seedling and plant growth when inoculated with the *B. siamensis* strain BW under salinity. Qi et al. [50] reported a similar scenario on cucumber growth parameters when inoculated with the *B. licheniformis* and *B. subtilis* harboring ACC (1-Aminocyclopropane-1-Carboxylate) Deaminase activity in saline stress conditions. Mohamed and Gomaa [51] claimed that *B. subtilis*, capable of increasing plant growth under saline stress, can be attributed to phytohormones and siderophore production. Din et al. [52] noted in their research that bacterial strains of the *Bacillus* genus alleviated salinity stress in wheat plants due to exopolysaccharide production and ACC deaminase activity. In another study, *B. pumilus* alleviated salt stress in wheat plants [53]. *Bacillus* strains increased rice growth in saline conditions by improving potassium and calcium ion acquisitions [54]. These findings are in accordance with previous studies in that the symbiosis of *B. aryabhattai* Z-48 and *B. subtillis* Z-12 substantially improved the growth attributes of turnip plants during saline stress. This can be attributed to the fact that these bacterial strains could be harboring plant-beneficial traits, e.g., ACC deaminase activity, mineral solubilization, and phytohormone production. In this study, *B. aryabhattai* strain Z-48 showed promising results in comparison to *B. subtillis* Z-12, which was also quite eminent. This variation in performance could be due to the different colonization potential of both bacterial strains and their varying plant-beneficial properties.

One essential physiological function of plants is photosynthesis, which sustains growth and increases resistance to environmental stressors [55]. In our research, the salt stress decreased photosynthetic pigments, including chlorophyll and carotenoids. This can be due to the increased chlorophyllase activity in salinity stress that leads to reduced photosynthetic efficiency [56]. Root-colonizing rhizobacteria increase iron availability to plants through siderophore activity, which increases chlorophyll content [57]. This, in turn, boosts photosynthetic activity and overall plant growth. This study observed the same as the application of *B. aryabhattai* Z-48, which helped plants increase chlorophyll production, leading to increased growth and performance under salinity stress. Previous studies have also demonstrated that PGPR inoculation increases chlorophyll content under salt stress. According to Khan et al. [58], inoculating rice with rhizospheric *Bacillus* strains under salt stress increases the amount of both chlorophyll a and b along with carotenoid contents. Similarly, in our study, the application of *B. aryabhattai* Z-48 could be attributed to increased chlorophyll production in turnip plants.

Salinity stress can affect the production of numerous bioactive phytochemicals, including glucosinolate, phenolic, and flavonoid compounds [59]. Some studies have depicted that salt stress increases the glucosinolate content, as salinity damages vacuoles from the point where these compounds are released [60]. Secondly, the hydrolyzed products of glucosinolates increase tolerance to abiotic and biotic stresses in plants [60]. As we observed, the application of bacterial strains, especially *B. aryabhattai* Z-48, further triggered the production of glucosinolates in turnip plants, which would have helped to ameliorate the salinity stress. Similarly, phenolic compounds are essential for detoxifying peroxides, quenching singlet oxygen, and absorbing and neutralizing free radicals [61]. Under salt stress, the turnip plants exhibited lower production of phenolic compounds. The same was observed in a previous study performed by Šamec et al. [62], where the onset of salinity stress (200 mM NaCl) decreased the production of phenolic contents in Chinese kale and white cabbage plants. Flavonoid compounds support defense mechanisms and control cellular activity. Under salt stress, flavonoids have been thought to function as chelators [63]. When *B. aryabhattai* Z-48 and *B. subtilis* Z-12 were applied, an elevated concentration of phenolic and flavonoid compounds was observed, which would have played their role in detoxifying reactive oxygen moieties and restoring ionic balance in turnip plants.

Exposure to salt results in the formation of reactive oxygen species, which severely damage proteins, lipids, and nucleic acids [64]. Plant protection against oxidative stress mainly depends on antioxidant mechanisms [65]. The superoxide anion is dismutated to H_2_O_2_ by superoxide dismutase, as the name of this enzyme indicates [66]. APX is an abundantly found antioxidant enzyme that scavenges H_2_O_2_ and reduces cellular damage [67]. CAT is crucial in detoxifying and reducing the amount of H_2_O_2_ in peroxisomes [68]. Plants’ ability to tolerate salt stress is thought to be enhanced by PGPR-induced antioxidative enzymes [69]. In this study, the antioxidant enzyme activities were relatively higher in plants receiving the inoculation of bacterial strains. Significant differences were observed in *B. subtilis* Z-12- and *B. aryabhattai* Z-48-treated plants under salinity or normal conditions compared with the respective controls. Similarly, in a previous study, the antioxidant system of rice was reported to be strengthened against salinity stress by applying *Bacillus* strains compared to the control plants [49].

## 5. Conclusions

The utilization of beneficial microbes is a sustainable and environmentally friendly approach for improving plant development in organic farming practices. *B. aryabhattai* Z-48 effectively rescued turnip plants against salinity stress by multiple mechanisms. This strain increased plant growth and development under both salinity and normal conditions and enhanced the production of photosynthetic pigments, glucosinolates, carotenoid contents, and the up-regulation of antioxidant machinery. This bacterial strain has the potential to be used in biofertilizer formulations.

## Figures and Tables

**Table 1:** Effect of PGPR and salinity stress on turnip plant chlorophyll, carotenoid, and total soluble sugar contents.

Treatment	Chl a (mg g^−1^ FW)	Chl b (mg g^−1^ FW)	Total Chl (mg g^−1^ FW)	Carotenoids (mg g^−1^ FW)	TSS (mg g^−1^ FW)
Control	0.88 ± 0.04 c	0.58 ± 0.08 c	1.46 ± 0.14 c	0.79 ± 0.04 b	1.74 ± 0.23 c
SS	0.43 ± 0.05 d	0.37 ± 0.01 d	0.80 ± 0.03 d	0.31 ± 0.013 d	1.19 ± 0.11 d
Z-12	1.68 ± 0.07 b	0.75 ± 0.04 bc	2.43 ± 0.4 b	0.81 ± 0.05 ab	2.32 ± 0.35 ab
Z-48	2.24 ± 0.13 a	1.20 ± 0.16 a	3.44 ± 0.57 a	1.05 ± 0.06 a	2.51 ± 0.18 a
SS + Z-12	0.77 ± 0.02 c	0.65 ± 0.05 c	1.42 ± 0.14 c	0.46 ± 0.03 c	1.55 ± 0.16 cd
SS + Z-48	1.72 ± 0.11 b	0.91 ± 0.08 b	2.63 ± 0.39 ab	0.59 ± 0.07 c	2.17 ± 0.29 b

Data are presented as mean ± standard error. The treatments followed by the same letter are not significantly different at *p* < 0.05. SS = salinity stress, Z-12 = *B. subtilis* strain Z-12, and Z-48 = *B. aryabhattai* strain Z-48.

**Table 2:** Effect of PGPR and salinity stress on SOD, POX, CAT, and APX enzyme activity of turnip plants.

Treatment	SOD (Units g^−1^ FW)	POX (µmol min^−1^ g^−1^ FW)	CAT (µmol min^−1^ g^−1^ FW)	APX (mmol min^−1^ g^−1^ FW)
Control	6.7 ± 0.98 d	1.94 ± 0.24 d	139 ± 9 d	2.1 ± 0.17 d
SS	12.4 ± 0.54 c	3.86 ± 0.57 cd	156 ± 13 c	3.78 ± 0.42 cd
Z-12	12.9 ± 0.47 c	5.75 ± 0.32 c	143 ± 12 cd	4.09 ± 0.33 c
Z-48	13.8 ± 0.63 b	8.12 ± 0.83 b	172 ± 11 a	4.47 ± 0.37 bc
SS + Z-12	14.6 ± 0.95 b	6.33 ± 0.52 bc	155 ± 14 c	5.77 ± 0.49 b
SS + Z-48	17.7 ± 1.2 a	9.78 ± 0.76 a	168 ± 10 b	6.25 ± 0.68 a

Data are presented as mean ± standard error. The treatments followed by the same letter are not significantly different at *p* < 0.05. SS = salinity stress, Z-12 = *B. subtilis* strain Z-12, and Z-48 = *B. aryabhattai* strain Z-48.

## Data Availability

The original contributions presented in this study are included in the article. Further inquiries can be directed to the corresponding author.
